# Functional Characterization of Chemosensory Proteins in the Scarab Beetle, *Holotrichia oblita* Faldermann (Coleoptera: Scarabaeida)

**DOI:** 10.1371/journal.pone.0107059

**Published:** 2014-09-04

**Authors:** Hongyan Sun, Li Guan, Honglin Feng, Jiao Yin, Yazhong Cao, Jinghui Xi, Kebin Li

**Affiliations:** 1 State Key Laboratory for Biology of Plant Diseases and Insect Pests, Institute of Plant Protection, Chinese Academy of Agricultural Sciences, Beijing, P.R. China; 2 College of Plant Science, Jilin University, Changchun, Jilin Province, P.R. China; University of Edinburgh, United Kingdom

## Abstract

Chemosensory proteins (CSPs) play important roles in chemical communication by insects, as they recognize and transport environmental chemical signals to receptors within sensilla. In this study, we identified *Hobl*CSP1 and *Hobl*CSP2 from a cDNA library of *Holotrichia oblita* antennae, successfully expressed them in *E. coli* and purified them by Ni ion affinity chromatography. We then measured the ligand-binding specificities of *Hobl*CSP1 and *Hobl*CSP2 to 50 selected ligands in a competitive binding assay. These results demonstrated that *Hobl*CSP1 and *Hobl*CSP2 have similar ligand-binding spectra. Both proteins displayed the highest affinity for β-ionone, α-ionone and cinnamaldehyde, indicating that they prefer binding to odorants other than sex pheromones. Additionally, immuno-localization revealed that *Hobl*CSP1 is highly concentrated in sensilla basiconica, while *Hobl*CSP2 is specifically localized to sensilla placodea. In conclusion, *Hobl*CSP1 and *Hobl*CSP2 are responsible for binding to general odorants with slightly different specificities due to their different in vivo environments.

## Introduction

The scarab beetle, *Holotrichia oblita* Faldermann (Coleoptera: Scarabaeida), is a prominent underground pest that causes great economic loss throughout its life cycle [1]. This insect is a polyphagous pest that feeds on a range of plants, such as peanut, soybean, wheat, and potato [2]. Studies have demonstrated that *H. oblita* responds differentially to different plant leaf odors, indicating that *H. oblita* can find hosts using plant volatiles as cues [3]–[5].

Insects develop an elaborate, sensitive, and specific olfactory system to perceive chemical signals in the environment. This system confers the capacity to communicate with mates, locate foods, oviposit, and avoid natural enemies [3], [6], [7]. The insect olfactory system is primarily composed of an antennae lobe in the brain and morphologically distinct sensilla [8]. Most sensilla locate in antennae and/or maxillary palps, which are rich in olfactory receptor neurons (ORN). Odorants pass through a specific channel in the cuticle to the ORN lymph, where they stimulate the odorant receptors (ORs). After the ORs are activated, the odorants are degraded by odorant-degrading enzymes (ODEs) [9].

Chemosensory proteins (CSPs), also known as olfactory specific-D like (OS-D like) proteins or sensory appendage proteins (SAPs), make up one of the most important sensor protein groups in insect chemoreceptors [10]. *Drosophila melanogaster* OS-D protein [11] and A-10 [12] were the first two reported CSPs. CSPs are generally acidic, soluble proteins that are approximately 13 kDa with 100–115 amino acids. All CSPs contain 4 conserved cysteine residues, which form 2 disulfide bonds (S-S) with their neighboring sulfur side chains. Each of these S-S bonds forms a ring, one by linking 8 surrounding amino acids, the other by linking 4 amino acids [13], [14]. Phylogenetic analysis of 180 CSPs from seven different insect orders demonstrated that CSPs are highly conserved with a N-terminal signature sequence, “YTTKYDN[VI][ND][LV]DEIL” [15], [16] and several α-helix domains in the secondary structures [17]–[20]. For example, *Bombyx mori* pheromone binding protein contains six α-helices, forming a cavity for binding pheromones [21]. The primary and secondary structures of CSPs are highly conserved across all insects [17], [19], [20]. This specific structure allows CSPs to interact with linear-chain compounds such as oleamide, which is an endogenous ligand of locust CSPs [22]. However, only a few three-dimensional CSP structures have been reported, including only *Mamestra brassicae* CSP-A6 (*Mbra*CSP-A6) [23], *Schistocerca gregaria* CSP4 (*Sgr*CSP4) [24], and *B. mori* CSP1 (*Bmor*CSP1) [25].

CSPs exist in both male and female organisms. They are not only found in sensilla within antennae [13], [26], [27], proboscises [28], maxillary palps [29], labial palps [30], and tarsus [31], but they are also observed in the abdomen, truncus, cuticle [32], legs [33], wings [22], and pheromone glands [34]. This non-confined localization would allow them to function in a wide spectrum of signaling events [35]. Furthermore, the expression of CSPs in insect pheromone glands indicates that these molecules are involved in formation and transportation of sex pheromones, such as lipocalin in the uterus and/or saliva of mammals [19]. The expression of *Sgre*CSP1-5 and *Mbra*CSP6 in the sensillum lymph indicates that they function in odorant sensing in the same manner as odorant binding proteins (OBPs) [36]. Additionally, differential expression of CSPs throughout development indicates that CSPs functions are also regulated temporally [37]. For example, *Apis mellifera* CSP5 (*Amel*CSP5) was found in the embryonic ectoderm of the Italian honeybee, a result was thought to be relevant to the embryo casings’ formation [38].

CSPs present in insects across different orders, including Diptera (4 CSPs in *D. melanogaster*, 8 CSPs in *Anopheles gambiae* [39]), Lepidoptera (10 CSPs in *M. brassicae* [28] and 16 CSPs in *B. mori*) and so on. Combining the recent discovery of 20 CSPs in *Tribolium castaneum,* these indicated the importance of CSPs and their potential as targets for pest control. Here, we constructed a cDNA library from antennae of *H. oblita* and found two CSPs, *Hobl*CSP1 and *Hobl*CSP2. Using a competitive binding assay with the fluorescent probe 1-NPN (N-phenyl-1-naphthylamine), we characterized the ligand-binding specificities and inspected the spatial localizations of these *Hobl*CSPs as a means to improve our understanding of CSPs roles in insects.

## Materials and Methods

### 1 Ethics statement

All animal experiments in this study were performed in strict accordance with the guidelines developed by the State Key Laboratory for Biology of Plant Diseases and Insect Pests, Institute of Plant Protection, and the Chinese Academy of Agricultural Science (IPP, CAAS). The protocol was approved by the committee on the Ethics of Animal Experiments of the IPP, CAAS. The Approval ID is SYXK (Beijing) and the Permit Number is 2008-008.

### 2 Insects

Adult scarab beetles (*H. oblita*) were collected from the Hefei Experimental Base of the Institute of Plant Protection, Anhui Academy of Agriculture Science, Hefei, Anhui Province, China, and maintained in the lab at 28°C under a standard photoperiod (L/D: 16 h/8 h). Antennae from adult females were excised and immediately frozen in liquid nitrogen.

### 3 Construction of an *H. oblita* antennae cDNA library

Total RNA from 100 female antennae was extracted with Trizol (Invitrogen, USA). Full-length double-stranded cDNA (ds cDNA) with blunt cDNA ends was synthesized and amplified using the Creator™ SMART™ cDNA Library Construction Kit (Clontech, USA). Synthesized ds cDNA was then incubated with 0.08 µg/µl proteinase K at 45°C for 20 min to inactivate the DNA polymerase. After size fractionation using CHROMA SPIN™ columns, the cDNA was incorporated into SfiI-digested λTripIE vector. The recombinant phage vector was transduced into *E. coli* XL1-Blue (TaKaRa Co., China). The plaques were counted to calculate the phage titer (pfu/ml), and the recombination efficiency was estimated by calculating the ratio of white (recombinant) to blue (non-recombinant) plaques. Fragments >350 bp were sequenced.

### 4 Identification and sequence analysis of *Hobl*CSP1 and *Hobl*CSP2

Using contig alignment coupled with NCBI BLAST, *Hobl*CSP1 and *Hobl*CSP2 were identified from the antennae cDNA library. The full-length sequences of *Hobl*CSP1 and *Hobl*CSP2 were cloned and verified by fishing with sequence-specific primers. The primers for *Hobl*CSP1 were: forward 5′-GAAAAGAAAAACGATAACGAA-3′ and reverse 5′-CACAATTTTACGTTGGAAGAT-3′, while the primers for *Hobl*CSP2 were: forward 5′-AGATATACAACAAAATATGATAA-3′ and reverse 5′-CAATGTATGCAACAGTGTCCAAG-3′. The signal peptides were predicted through SignalP 3.0 [40], and the molecular weights were calculated using the SWISS-PROT (ExPASy server) program “Compute pI/Mw.” The hydrophobicity of each predicted protein was analyzed at http://us.expasy.org/cgi-bin/protscale.pl. And, BLAST and Mult-Alin were used for homology searches and the alignment of nucleotide and/or amino acid sequences.

The evolutionary history of insect CSPs was inferred by using the Maximum Likelihood method based on the JTT matrix-based model [41] in MEGA 6.06 [42]. Briefly, the CSP cDNAs were aligned in ClustalW2 [43] and the alignment was improved by removing the spurious sequences and poorly aligned regions in TrimAl [44] by setting the gap threshold to 0.25. The tree with the highest log likelihood (−11948.7261) is shown. The analysis involved 109 amino acid sequences. All positions with less than 95% site coverage were eliminated. That is, fewer than 5% alignment gaps, missing data, and ambiguous bases were allowed at any position.

### 5 Prokaryotic expressions of *Hobl*CSP1 and *Hobl*CSP2

Recombinant pET30a(+)/CSP1 and pET30a(+)/CSP2 were generated by ligating the sticky ends of the designed *Hobl*CSP1 and *Hobl*CSP2 constructs into the expression vector pET30a (Novagen, Germany). pET30a(+)/CSP1 and pET30a(+)/CSP2 were then transformed into *E. coli* BL21 (DE3) pLysS cells. The expressions of recombinant *Hobl*CSP1 and *Hobl*CSP2 were induced for 4 h by 0.5 mM IPTG following a 3 h pre-incubation. The cells were harvested by centrifugation and then homogenized in phosphate-buffered saline (PBS, 0.04 M, pH 7.0). After centrifugation at 12,000×g for 20 min at 4°C, the supernatants were purified by Ni ion affinity chromatography (GE-Healthcare). Recombinant *Hobl*CSP1 and *Hobl*CSP2 were identified by western blot analysis with antibodies designed against the His-tag (Abcam, USA). To prevent confounding effects in the subsequent experiments, the His-tag was removed by recombinant enterokinase (rEK) (Bio Basic Inc.) and NaCl was removed by dissolving the proteins in dH_2_O and filtering with a 10 kDa Amicon Ultra-0.5 Device (Millipore, USA). Finally the recombinant proteins were stored at −80°C, until required.

### 6 Fluorescence competition assays

Fluorescence binding assay was based on method previously described by Yin et al. [45]. Briefly, fifty compounds (Sigma-Aldrich, Germany) with chemical purities ≥97% were tested in a Lengguang 970 CRT spectrofluorimeter (Shanghai Jingmi, China). Assuming the proteins were 100% active, the binding affinities for N-phenyl-1-naphthylamine (1-NPN) were determined by adding aliquots of 1-NPN into a 2 µM protein solution for final concentrations of 2∼24 µM. The dissociation constants of the binding competitors were calculated from IC_50_ according to Campanacci et al: Ki = [IC_50_]/(1+[1-NPN]/K_(1-NPN)_), where [1-NPN] represents the concentration of unbound 1-NPN and K_(1-NPN)_ is the dissociation constant of 1-NPN [46]. Binding data for each ligand was collected from 3 measurements.

### 7 Preparation of anti-*Hobl*CSP1 and anti-*Hobl*CSP2 antibodies

Purified full-length *Hobl*CSP1 and *Hobl*CSP2 were injected into New Zealand white rabbits following a standard immunization protocol for antibody production. Briefly, 100 µg of recombinant CSP was injected with an equal volume of Freund’s complete adjuvant, followed by three additional injections of 500 µg, with one each on the 21st, 35th, and 49th day. The antiserum was then tested using an enzyme-linked immunosorbent assay (ELISA) and used without further purification. The pre-injected rabbit serum was used as a negative control.

### 8 Spatial localizations of *Hobl*CSP1 and *Hobl*CSP2 in antennae of *H. oblita*

Antennal lamellae from both males and females were excised from adult *H. oblita* and fixed in a mixture of 4% paraformaldehyde (Thermo Scientific, USA) and glutaraldehyde (2%) in 0.1 M PBS (pH 7.4). They were then embedded in LR White resin (TAAB, UK) after dehydration in a graded ethanol series. Ultrathin sections (500–700 nm) were cut on a microtome with glass blades and then incubated with primary anti-*Hobl*CSP1 (1∶2000) and anti-*Hobl*CSP2 (1∶2000) antibodies at 4°C overnight. After incubation, the sections were washed in 2 times in PBGT then incubated with anti-rabbit IgG secondary antibody (1∶20), coupled to 10-nm colloidal gold, for 90 min at room temperature. Gold granules were size-increased by silver intensification for 15 min in the dark, followed by incubation with 2% uranyl acetate for 15 min to increase the contrast. The samples were imaged by transmission electron microscopy (HitachiH-7500).

## Results

### Characteristics of the *Hobl*CSP1 and *Hobl*CSP2 sequences

**Figure 1:**
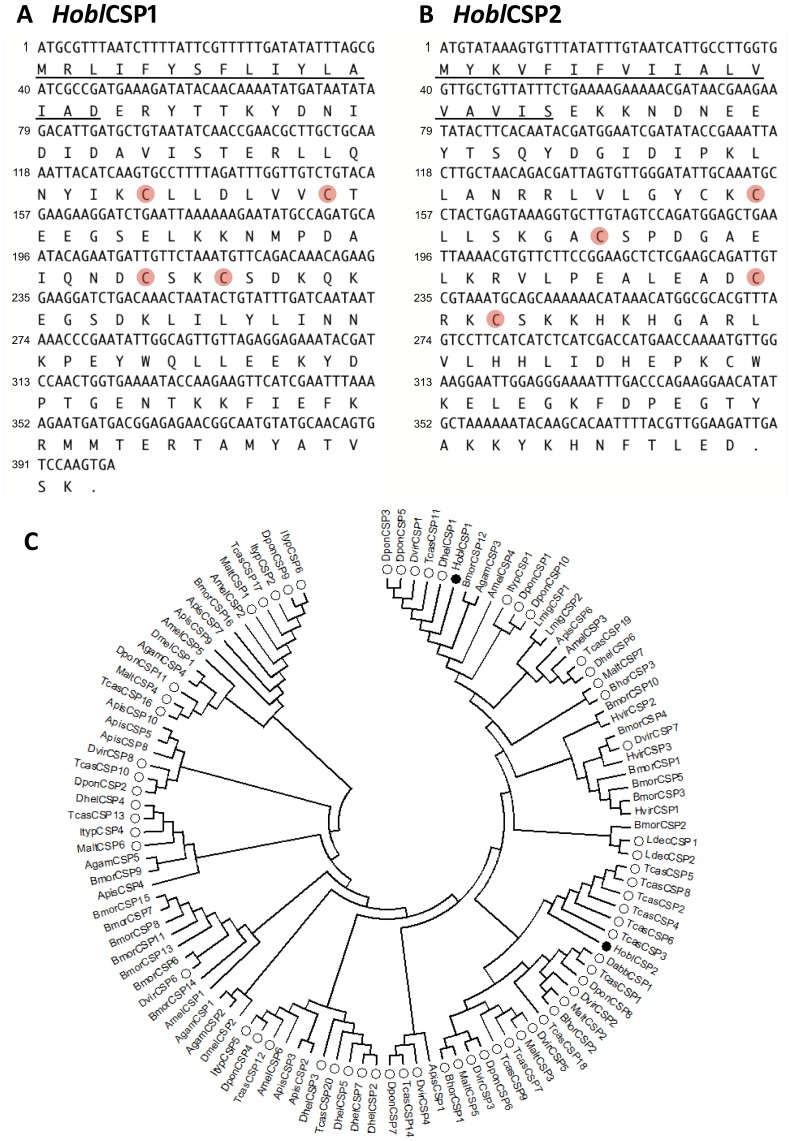
Characterization and phylogenetic tree of *Hobl*CSP1 and *Hobl*CSP2. (**A–B**) Nucleotide and putative amino acid sequence analysis of *Hobl*CSP1 (**A**) and *Hobl*CSP2 (**B**). The predicted signal peptides are underlined (Generated from: http://bioinformatics.leeds.ac.uk/prot_analysis/Signal.html). The four conserved cysteine residues are highlighted in pink, and the stop codons are marked as dots in both sequences. (**C**) Molecular phylogenetic analysis by Maximum Likelihood method. CSPs used include *Hobl*CSP1, *Hobl*CSP2 and 20 CSPs from *T. castaneum*, 11 from *D. ponderosae* [53], 6 from *Ips typographus* [53], 8 from *Diabrotica virgifera* [51], 1 from *D. abbreviates* [51], 2 from *L. decemlineata* [51], 7 from *M. alternatus* [52], 7 from *Dastarcus helophoroides* [52], 3 from *B. horsfieldi* [50], 16 from *B. mori* [69], 3 from *Heliothis virescens* [70], 6 from *Apis mellifera* [16], 2 from *Locusta migratoria* [22], 5 from *A. gambiae* [57], 2 from *D. melanogaster* [15], 10 from *Acyrthosiphon pisum* [71]. *Hobl*CSP1 and *Hobl*CSP2 are marked with solid dark circles and all other CSPs from Coleopteran are marked with open circles. All sequences are available from the NCBI database.

From the antennae cDNA library, full-length *Hobl*CSP1 (GenBank: HQ683720) and *Hobl*CSP2 (GenBank: HQ688991) genes were obtained and verified. The open reading frame (ORF) of *Hobl*CSP1 contained 399 nucleotides, encoding 132 amino acids. The predicted molecular weight of *Hobl*CSP1 was 15.55 kDa. The ORF of *Hobl*CSP2 was comprised of 390 nucleotides, encoding 129 amino acids, and the predicted molecular weight was 14.78 kDa. At their N-termini, *Hobl*CSP1 and *Hobl*CSP2 contain signal peptides of 16 and 18 residues, respectively. Both proteins contained 4 conserved cystine residues (Fig. 1A, 1B), consistent with the model Cys-X_6–8_-Cys-X_16–21_-CysX_2–4_-Cys, and also contained a hydrophobic domain. The isoelectric points (PI) of *Hobl*CSP1 and *Hobl*CSP2 were dramatically different at 4.93 and 8.14, respectively. Phylogenetic analysis based on the deduced amino acid sequence also revealed that *Hobl*CSP1 and *Hobl*CSP2 are highly divergent, with *Hobl*CSP1 and *Hobl*CSP2 in two separated Coleopteran mono-phylogenetic groups (Figure 1C).

### Procaryotic expression of *Hobl*CSP1 and *Hobl*CSP2

**Figure 2:**
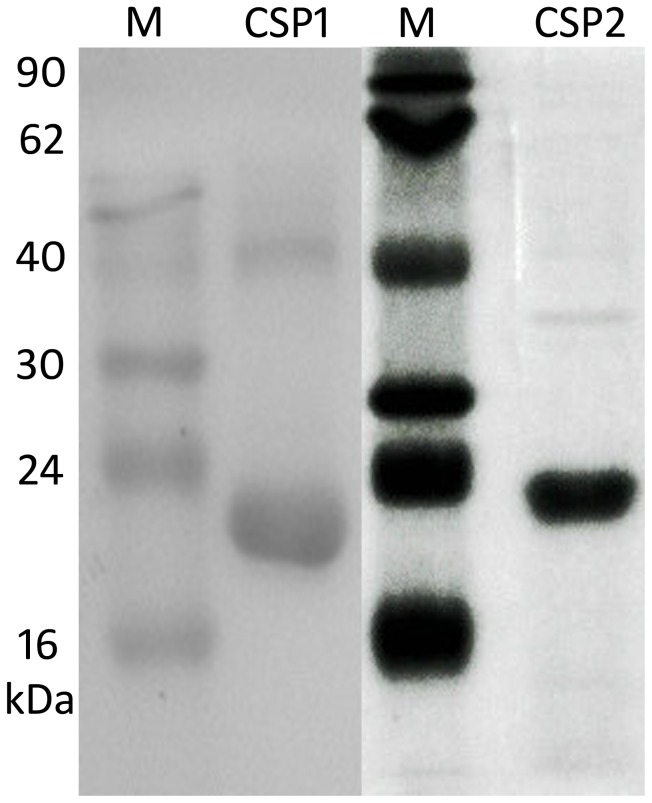
Prokaryotic expression and purification of *Hobl*CSP1 and *Hobl*CSP2. The purified fusion proteins pET30a(+)/CSP1 and pET30a(+)/CSP2 are shown in separate lanes labeled CSP1 and CSP2, respectively. M: protein molecular weight marker (top to bottom: 90, 62, 40, 30, 24, 16 kDa). *Hobl*CSP1 and *Hobl*CSP2 were identified by using antibodies designed against the His-tag.

The recombinant proteins pET30a(+)/CSP1 and pET30a(+)/CSP2 were successfully expressed in BL21(DE3) PlysS cells. For both CSPs, a specific band less than 24 kDa (including His-tag) was observed by western blot analysis (Figure 2), which was consistent with the molecular weight deduced from their predicted amino acid sequences. *Hobl*CSP1 and *Hobl*CSP2 were purified at concentrations of 1.1 mg/ml and 1.2 mg/ml, respectively.

### Binding specificities of *Hobl*CSP1 and *Hobl*CSP2 largely overlapped

**Figure 3:**
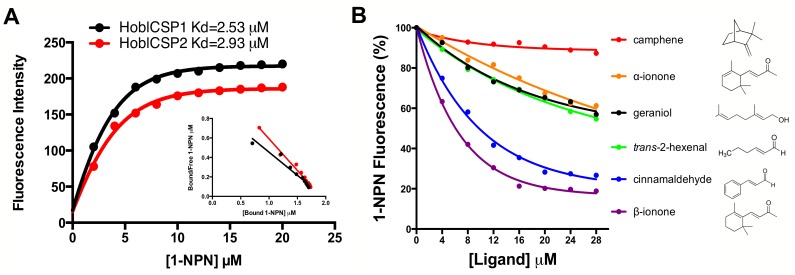
Fluorescence competition assay of the recombinant *Hobl*CSP1 and *Hobl*CSP2. (**A**) Binding curves of 1-NPN with *Hobl*CSP1 and *Hobl*CSP2 and their relative Scatchard plots. The dissociation constant of *Hobl*CSP1 is 2.53 µM and that of *Hobl*CSP2 is 2.93 µM. (**B**) Representative competitive binding curves of the recombinant *Hobl*CSP1 to a series selected ligands (see Table 1). All proteins used were diluted to a fixed concentration of 2 µM, while the concentration of 1-NPN varied with the respective dissociation constant of *Hobl*CSPs/1-NPN. The mixed solution was then titrated with 1 mM of each competing ligand to final concentrations of 0–28 µM. Fluorescence intensities are plotted as percent of the initial fluorescence in the absence of ligands. The molecular formulas of representative ligands are shown here as well. The calculated dissociation constants for all of the ligands are listed in Table 1.

Based on the dissociation constants of CSP1/1-NPN (2.53 µM) and CSP2/1-NPN (2.93 µM) calculated from the binding curves (Figure 3A), fifty potential odorant compounds were selected for a fluorescence competition assay with 1-NPN. These molecules included *Ricinus communis* leaf volatiles that attract *H. oblita* [47]; volatiles isolated from *H. oblita* host plant, *Ulmus pumila* Linnaeus [48], [49]; putative sex pheromones from closely related beetles; and previously reported compounds (Table 1). The inhibition constants Ki (for each CSP/ligand combination) are summarized in Table 1. The binding curves of a few representative fluorescence competition assays are presented in figure 3B. These binding curves, coupled with the Ki values, demonstrated that *Hobl*CSP1 and *Hobl*CSP2 displayed similar spectra of binding activity. Of the 50 selected compounds, *Hobl*CSP1 preferred 22, while *Hobl*CSP2 preferred 18. Within these groups, 15 compounds were covered in the binding spectra of both *Hobl*CSP1 and *Hobl*CSP2 (Table 1). Furthermore, both *Hobl*CSP1 and *Hobl*CSP2 bound most strongly to β-ionone, followed by α-ionone and cinnamaldehyde. Other ligands, however, were unique to each of the proteins. For example, *Hobl*CSP1 was able to bind camphene, albeit with a high K_i_ value, while *Hobl*CSP2 could not bind camphene at all (Figure 3B).

**Table 1:** Affinities of selected *Hobl*CSP1 and *Hobl*CSP2 pure odorant ligands.

Ligands	Purity (%)	*Hobl*CSP1 *Ki*	*Hobl*CSP2 *Ki*
**Green leaf volatiles**			
hexanol	≥99	57.64	60.53
cis- 3- hexen-1-ol	≥98	–	47.84
cis-3-hexen-1-ol	95	46.89	35.68
4-tert-butylcyclohexanol	≥96	34.60	50.68
trans-2-hexenal	98	24.89	25.65
2-Ethy1-1- Hexanol*	≥98	29.85	32.07
**Sex pheromones**			
Glycine ethyl ester	98	43.47	39.48
L-Isoleucine methyl este	95	N	N
L-Proline methyl ester	95	56.97	–
**Aldehydes compounds**			
1-Heptaldehyde	≥95	N	N
octylaldehyde*	99	N	N
decanal*	97	N	N
nonanal*	95	N	N
1-Octen-3-ol	98	48.08	–
2-Tridecanone		N	N
6-Methyl-5-hepten-2-one*	99	–	56.91
1-octanol	99	35.89	40.65
**Alkanes compounds**			
hexane	99	N	N
n-Undecane	99	N	N
tetradecane	99.8	N	N
pentadecane	99.8	N	N
hexadecane	98	N	N
methyl palmitate	97	–	–
n-Hexadecane	99	N	N
**Aromatic compounds**			
benzyl alcohol	99	34.89	54.78
benzaldehyde	≥99.5	23.35	19.47
cinnamaldehyde	≥93	20.48	17.49
anisole	≥95	28.36	29.47
dimererhyl phthalate	≥99.5	22.27	–
eugenol	99	30.01	–
methyl salicylate	99	47.34	–
methyl anthranilate	≥98	N	N
**Terpenoids**			
limonene	97	–	–
α-ionone	90	10.53	11.45
β-ionone	90	4.30	4.47
phellandrene	≥95	N	N
octadecene	90	N	N
myrcene	≥95	25.36	29.48
camphene*	95	37.92	–
camphor	96	N	N
α-pinene*	99+	–	–
β-pinene*	99+	–	–
nerolidol	98	N	N
β-caryophyllene*	≥98	N	N
retinol	98	N	N
linalooloxide	≥97	–	–
L-(−)-Linalool	≥98.5	–	–
geraniol	98	29.45	30.24
α -terpineol	≥96	46.83	39.43
**Heterocyclic compound**			
indole*	≥99	–	–

“–” represents ligands whose IC_50_ exceeded 100 mM.

“N” represents no binding activity at all.

“*” represents plant volatiles from *Ulmus pumila.*

### Spatial localizations of *Hobl*CSP1 and *Hobl*CSP2 in antennae of *H. oblita*

**Figure 4:**
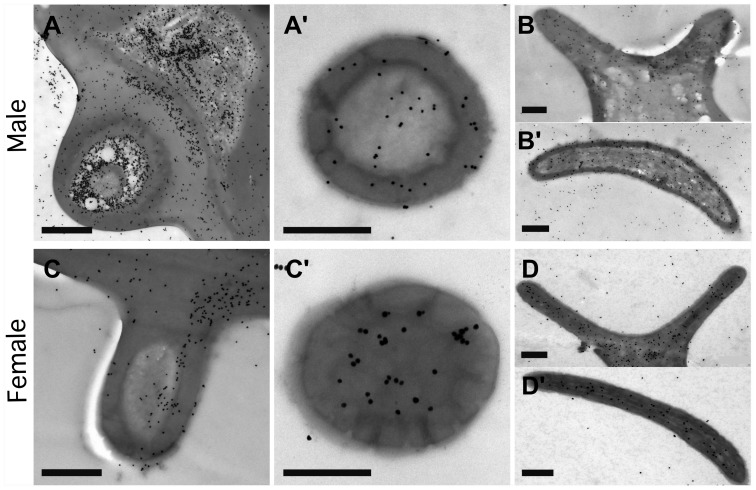
Spatial localization of *Hobl*CSP1 in olfactory sensilla basiconica and sensilla placodea of adult *H. oblita* antennae. All samples are labeled with an anti-*Hobl*CSP1 antibody. *Hobl*CSP1 proteins are shown as black dots. (**A–A’**) Longitudinal (**A**) and Cross (**A’**) sections of male sensilla basiconica; (**B–B’**) Longitudinal (**B**) and Cross (**B’**) sections of male sensilla placodeum; (**C–C’**) Longitudinal (**C**) and Cross (**C’**) sections of female sensilla basiconica; (**D–D’**) Longitudinal (**D**) and Cross (**D’**) sections of female sensilla placodeum. All antibodies used were diluted to 1∶5000. Each treatment was repeated more than 3 times. Scale bar = 500 nm.

**Figure 5:**
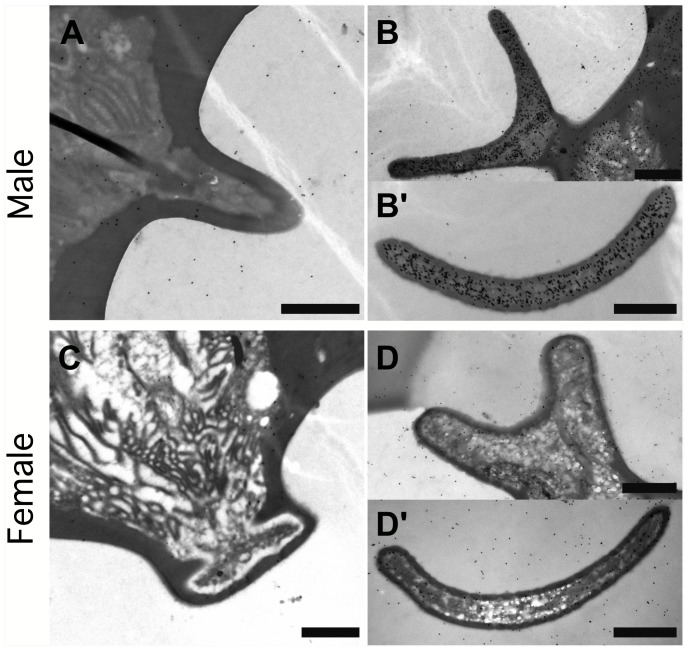
Spatial localization of *Hobl*CSP2 in olfactory sensilla basiconica and sensilla placodea of adult *H. oblita* antennae. All samples are labeled with an anti-*Hobl*CSP2 antibody. *Hobl*CSP2 proteins are shown as black dots. (**A**) Longitudinal section of male sensilla basiconica; (**B–B’**) Longitudinal (**B**) and cross (**B’**) sections of male sensilla placodeum; (**C**) Longitudinal section of female sensilla basiconica; (**D–D’**) Longitudinal (**D**) and cross (**D’**) sections of female sensilla placodeum. All antibodies used were diluted to 1∶10000. Each treatment was repeated more than 3 times. Scale bar = 1 µm.

Generally, both *Hobl*CSP1 and *Hobl*CSP2 were found in the antenna of both male and female adult *H. oblita*. These two proteins were primarily distributed in the outer sensillum lymph, with different concentrations in different types of sensilla. *Hobl*CSP1 was concentrated in sensilla placodea of both males and females (Figure 4B, 4B’, 4D and 4D’), and more highly concentrated in male sensilla basiconica (Figure 4A, 4C). However, *Hobl*CSP2 was found to be sensilla-specific, as it was highly expressed in sensilla placodea but rarely found in sensilla basiconica (Figure 5).

## Discussion

In this study, we characterized two CSPs, *Hobl*CSP1 and *Hobl*CSP2, from a *H. oblita* antennal cDNA library. Similar numbers of CSPs were also found in other beetles, like 3 CSPs in *Batocera horsfieldi* [50], 2 CSPs in *Leptinotarsa decemlineata*, and 1 CSP in *Diaprepes abbreviatus* [51]. In contrast, 12, 11, and 20 CSPs were found in *Monchamus alternatus* [52], *Dendroctonus ponderosae* [53], and *T. casteneum* [54] respectively. Different numbers of CSPs may be required to distinguish different host plants due to the varieties of their ligand-binding specificities. However, further investigations are required to verify this. *Hobl*CSP1 and *Hobl*CSP2 are highly divergent in deduced amino acid sequences. Additionally, phylogenetic analysis demonstrated that *Hobl*CSP1 and *Hobl*CSP2 are not in a recent mono-phylogeny group (Figure 1C), indicating that they diverged from each other a long time ago. Coupled with the markedly different PIs of *Hobl*CSP1 (4.93) and *Hobl*CSP2 (8.14), we predicted that *Hobl*CSP1 and *Hobl*CSP2 function differently. Surprisingly, we found that the binding spectra of *Hobl*CSP1 and *Hobl*CSP2 largely overlapped. Among the 50 selected compounds, β-ionone and its isoform α-ionone and cinnamaldehyde displayed the highest affinities to both *Hobl*CSP1 and *Hobl*CSP2. This is consistent with the result from the electroantennogram (EAG) examinations that showed α-ionone and cinnamaldehyde can elicit strong electrophysiological responses in *H.oblita* antennae [1]. Also, a previous study showed cinnamaldehyde displays a great attraction to *H.oblita* [55]. β-ionone and α-ionone are general plant volatiles [56] that are found in *H.oblita*’s host plant, potato [2], while cinnamaldehyde is a main volatile from *H.oblita*’s host plant, castor [2], [55]. These three chemicals showed the best affinities to *Hobl*CSPs, illustrating the underlying mechanism via which *H.oblita* recognizes their host plants. These chemicals would be the best candidates to distract *H.oblita* from recognizing their host plants, which in turn can be applied to successfully control them in the field.

Also, *Hobl*CSP1 and *Hobl*CSP2 exhibited medium affinities to *trans*-2-hexenal, geraniol, myrcene, and benzaldehyde, and limited affinities to sex pheromones such as L-proline methyl ester, Glycine ethyl ester, and L-isoleucine methyl ester. Both proteins bound to very few alkanes, alcohols, and aldehydes.

These data demonstrated the discriminatory power of insect olfactory systems, with the ability to distinguish different isomers of the same compound [45]. Particularly, *Hobl*CSP1 and *Hobl*CSP2 preferred benzene rings in a ligand structure (Figure 3B). These preferential binding affinities of *Hobl*CSP1 and *Hobl*CSP2 indicated that they play important roles in odorant binding beyond simply the sex pheromone response, although *Hobl*CSP1 and *Hobl*CSP2 are also found in locations other than the antennae (unpublished data).

However, *Hobl*CSP1 and *Hobl*CSP2 reserved unique binding affinities to other compounds. *Hobl*CSP1 displayed higher affinities to aromatic compounds, including dimererhyl phthalate, eugenol, and methyl salicylate, whereas *Hobl*CSP2 showed higher affinities to green leaf volatiles, such as *cis*-3-hexen-1-ol, cis-3-hexen-1-ol, and 6-methyl-5-hepten-2-one. Since the binding affinities of *Hobl*CSP1 and *Hobl*CSP2 were tested under the same condition, it is therefore possible that they may display different binding activities due to their different in vivo environments. Interestingly, the homologous CSPs in different species could also have divergent affinities. In our experiments, the best ligand of *Hobl*CSP1 is β-ionone, whereas the best ligands for its homologous *Agam*CSP3 are 2-pentylcinnamaldehyde, retinal, citronellal, and nonanal [57]. This could be due to the adaptation of insect olfactory systems to the specific odorant of their hosts [58]–[61].

*H. oblita* antennae are sexually dimorphic. The sensilla placodea and sensilla basiconica are the most common sensilla in the antennae of beetles [62]. The numbers of sensilla placodea and sensilla basiconica are approximately equal in females, whereas in males there are significantly more (9 times) sensilla basiconica than sensilla placodea. However, their functions remain unknown. The sensilla placodea, rather than sensilla basiconica, was proposed as the organ responsible for responding to sex pheromones in *Popillia japonica* and *Anomala osakana* [63]. In *Anomala corpulenta*, the sensilla diverged for different functions, with the umbilicate sensilla placodea responding to green leaf volatiles and the hidden sensilla placodea primarily interacting with sex pheromones [64]. In *H. oblita,* scientists have proposed that the sensilla basiconica is the sensillum used for sex pheromone responses [65] because females synthesize sex pheromones and males respond to them, most likely through CSPs in the antennae [66], [67]. Here, we found that *Hobl*CSP1 was highly concentrated in the sensilla basiconica, and *Hobl*CSP2 was densely localized in the sensilla placodea. This result indicated that, due to its higher affinity to odorants, *Hobl*CSP1 may confer sensilla basiconica the ability to respond to sex pheromones. Additionally, the localizations of *Hobl*CSPs excluded the possibility that they function as homo or heterodimers, which is consistent with a previously published fluorescence competition assay [48].

CSPs closely relate to insect behavioral plasticity. Our work here discovered that *Hobl*CSP1 and *Hobl*CSP2 have specialized characteristics and unique localization patterns. These will assist in devising strategies to disrupt the aggregation of *H. oblita*. Also, 924 OBPs and 300 CSPs had been identified (UniProt) to date [68], especially up to 20 CSPs in a single species [54]. The ligand binding overlap between different CSPs, and between CSPs and OBPs, point to intriguing questions regarding the evolution of insect olfactory systems and the underlying mechanisms of olfactory recognition.
